# Customised 3D-Printed Surgical Guide for Breast-Conserving Surgery after Neoadjuvant Chemotherapy and Its Clinical Application

**DOI:** 10.3390/bioengineering10111296

**Published:** 2023-11-09

**Authors:** Jie Luo, Feng Chen, Hong Cao, Wei Zhu, Jian Deng, Dan Li, Wei Li, Junjie Deng, Yangyan Zhong, Haigang Feng, Yilin Li, Xiongmeiyu Gong, Jutao Zeng, Jiaren Chen

**Affiliations:** 1 Department of Breast and Thyroid Surgery, Clinical Research Center for Breast & Thyroid Disease Prevention in Hunan Province (2018SK4001), The Second Hospital, University of South China, Hengyang 421001, China; 2 National Engineering Research Centre for High Efficiency Grinding, College of Mechanical and Vehicle Engineering, Hunan University, Changsha 410082, China

**Keywords:** breast cancer, surgery guide, 3D printing, neoadjuvant chemotherapy, preoperative planning, breast-conserving surgery, biomedical devices

## Abstract

For patients eligible to undergo breast-conserving surgery (BCS) after neoadjuvant chemotherapy, accurate preoperative localisation of tumours is vital to ensure adequate tumour resection that can reduce recurrence probability effectively. For this reason, we have developed a 3D-printed personalised breast surgery guide (BSG) assisted with supine magnetic resonance imaging (MRI) and image 3D reconstruction technology, capable of mapping the tumour area identified on MRI onto the breast directly using dual positioning based on the manubrium and nipple. In addition, the BSG allows the colour dye to be injected into the breast to mark the tumour region to be removed, yielding more accurate intraoperative resection and satisfactory cosmetic outcomes. The device has been applied to 14 patients from January 2018 to July 2023, with two positive margins revealed by the intraoperative biopsy. This study showed that the BSG-based method could facilitate precise tumour resection of BCS by accurately localising tumour extent and margin, promoting the clinical efficacy in patients with breast cancer as well as simplifying the surgical process.

## 1. Introduction

Breast cancer has surpassed lung cancer as the most diagnosed cancer worldwide (approximately 2.3 million in 2020), accounting for 24.5% of all cancer cases [1]. For early breast cancer, breast-conserving surgery (BCS) has become the preferred surgical treatment strategy, while locally advanced breast cancer is treated generally via BCS after neoadjuvant chemotherapy (NACT) that can downstage the tumour as well as reduce tumour size [2,3]. However, NACT can cause the original tumour to shrink or scatter remarkably, imposing a significant challenge on the preoperative localisation of residual tumour extent, thus leading to intraoperative incomplete or excessive tumour resection [4,5,6]. Inadequate resection increases the probability of recurrence and reoperation, whilst excessive resection deteriorates cosmetic outcomes. Consequently, accurate preoperative localisation of residual tumour extent and margin after the treatment of NACT is of great importance to patients eligible to undergo BCS, particularly those with locally advanced breast cancers.

Of many imaging modalities commonly employed to determine tumour extent and location, such as computed tomography, mammography, sonography and magnetic resonance imaging (MRI), MRI is considered more suitable for identifying residual tumours after NACT due to its higher accuracy [5,7,8]. A vital difficulty of BCS is how to accurately map the digitalised tumour information acquired from the aforementioned medical imaging methods onto the patient’s breast. To address this issue, various surgical guidance devices, including wire-guided localisation, clip marker insertion and radio-guided localisation, have been developed for aiding preoperative breast localisation and surgical excision [9,10,11,12]. However, each localisation technique above possesses its own clinical limitations due to intrinsic disadvantages in aspects of localisation accuracy, procedural complexity, invasiveness to the patient and safety, and a detailed review is available from Corsi et al. [13,14,15]. The wire-guided methods can cause pain and bleeding, thus making them more invasive and less user-friendly; the radio-based technique requires strict nuclear regulatory protocols and multiple processes for manipulating radioactive seeds, complicating the surgical procedure dramatically. Non-wire nonradioactive devices are, therefore, more suitable alternatives to mark the tumour extent for precise surgical resection [13].

The emergence of three-dimensional (3D) printing has advanced the capability to fabricate 3D biomedical devices with architecturally complex features, such as biomimetic implant scaffolds [16,17], microneedles [18] and recently surgery guide devices [19,20,21,22,23]. Cappello et al. presented a workflow to produce a patient-specific 3D-printed model for pre-surgical planning of cardiac surgery, which can help surgeons to locate stenosis and the point for bypass placement [22]. For BCS, Barth et al. demonstrated that the location and shape information extracted from a supine MRI could be projected safely to a patient’s breast using a 3D-printed form, lowering the rate of positive margins compared with the wire-guided method [19]. Afterwards, Wu et al. developed a 3D-printed breast surgical guide (BSG) based on prone and supine MRI to locate the target tumour area. The device was tested on one patient admitted to BCS after NACT, and the tumour was removed safely, highlighting the feasibility of the surgical guide in facilitating precise tumour resection [20]. However, the current BSG employs a single-point positioning approach based on the nipple when attaching the device to the surface of the breast, bringing about inaccurate localisation due to rotation or deviation. Furthermore, a single specimen was adopted in the study by Wu et al. [20]; the reliability and reproducibility that are necessary for clinical application were not demonstrated.

This study proposes a customisable 3D-printed surgical guide based on supine MRI and image 3D reconstruction technology for breast-conserving surgery after NACT, followed by surgical verification and postoperative pathological assessments. First, the eligibility of patients for BCS was determined with detailed patient characteristics. The 2D supine MRI image slices were then reconstructed to represent 3D breast organs, within which the 3D tumour can be segmented. Based on the digital information of segmentation and reconstruction, a surgical guide was designed with the ability to position the device on the surface of the breast and locate the internal tumour extent accurately. Finally, the clinical practice was investigated by applying the device to 14 patients, followed by postoperative pathological analysis.

## 2. Materials and Methods

### 2.1. Inclusion Criteria

Patients who met the following criteria were admitted: (1) the patient was diagnosed with locally advanced invasive breast cancer confirmed by our hospital; (2) the patient completed NACT successfully and was willing to undergo BSC after NACT; (3) the shrinking pattern of the initial tumour after NACT was diagnosed to be concentric contraction [24]. Patients with inflammatory breast cancer, multicentric lesions and claustrophobia were excluded. Patients allergic to gadolinium contrast agents were not eligible. A total of 20 patients with locally advanced invasive breast cancer diagnosed by biopsy pathology were collected in the hospital from January 2018 to July 2023. Fourteen patients provided informed consent and agreed to undergo the supine magnetic resonance imaging and to use the surgical guide during the operation. This study was approved by the Ethics Committee Review Board of the hospital.

Detailed patient information, such as age, contact information, operation method, initial tumour diameter, tumour size after neoadjuvant chemotherapy and operation time, was recorded. The tumour characteristics are summarised in Table 1. The pathological indicators of breast cancer were determined by immunohistochemistry (IHC), including pathological type, histological grade, expression of oestrogen receptor (ER), progesterone receptor (PR) and Ki-67 index level. The expression of epidermal growth factor receptor-2 (HER-2) was judged by combining IHC and fluorescence with in situ hybridisation (FISH).

### 2.2. Design and Manufacturing of the Surgical Guide

**Figure 1:**
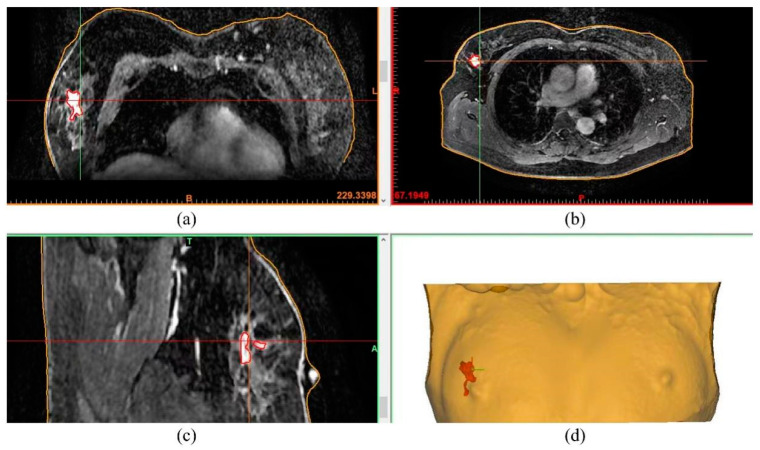
Supine MRI images showing the tumour’s location and margin marked as the red contours: (**a**) axial view; (**b**) coronal view; (**c**) sagittal view. (**d**) 3D model reconstruction of the tumour and surrounding tissues based on the MRI image series. The red patch depicts the tumour.

To minimise the localisation error, the supine position identical to the operation’s was adopted during MRI image acquisition. Breast MRI images were acquired using a 3.0 T MRI system (GE premier3.0T, Chicago, IL, USA) with plain scan AxT1, AxT2, Ax + Sag C+ and injection of 15 mL contrast agent (gadolinium diammonium, GE HealthCare, Chicago, IL, USA) at a rate of 2.5 mL/s, followed by rinsing with 10 mL of 0.9% normal saline (see Figure 1a–c). The patient was removed from the machine immediately after the final dynamic sequence.

**Figure 2:**
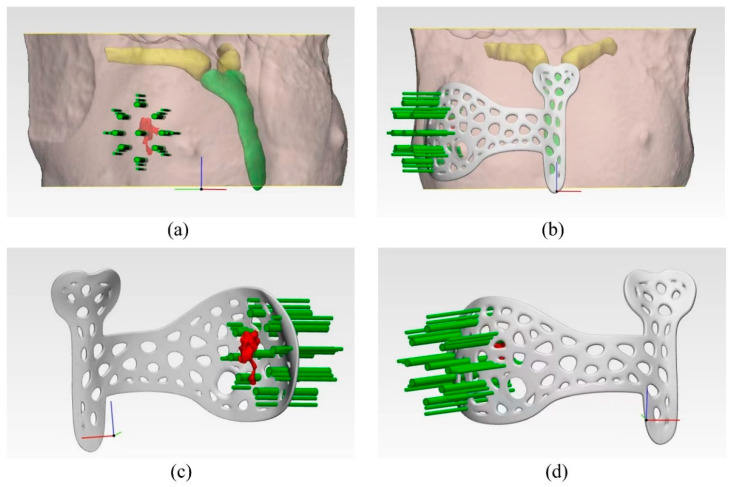
Design of the breast surgery guide based on the 3D segmented model that displays the tumour location and surrounding tissues: (**a**) Determination of tumour resection boundary. The middle point of the longest axis of the tumour was selected as the centre point of the tumour, based on which a circle indicated by green columns was drawn with a diameter slightly extended by 0.5 cm from the MRI tumour boundary to ensure a safe margin. (**b**) The shape of the surgery guide was tailored to fit precisely with that of the patient’s breast and surrounding tissues. A circular hole located at the nipple was generated as the main positioning point when the surgeon applied the device onto the surface of the breast. The manubrium was chosen as the second positioning point to promote localisation accuracy. (**c**) The tumour-side view of the surgery guide containing quantitative information about tumour extent and resection margin. (**d**) The front view of the surgery guide.

Based on the supine MRI image series (DICOM format), tumour segmentation was carried out slice-by-slice by our experienced radiologists using a digital medical design platform (FirePlus 3D v2.01, Copyright © 2022 BlackFlame Inc., Shanghai, China), as indicated by the red contours in Figure 1a–c. Segmentation was performed manually by using the thresholding-based method. Image reconstruction was then conducted using the same platform based on the MRI slices with segmentation marks to generate the 3D model, presenting the tumour location and extent in 3D space and other critical information such as the shape of the breast and manubrium (see Figure 1d and Figure 2). Subsequently, the breast surgery guide (BSG) was designed based on the 3D reconstruction model. The shape of the device was tailored precisely to match that of the patient’s breast and surrounding tissues. A circular hole that overlaps with the nipple was generated as the main positioning point when the BSG was applied to the surface of the breast. Except for the positioning point at the nipple, the location of the manubrium was also incorporated into the device (see Figure 2b). Such dual positioning ensures localisation accuracy by avoiding rotation during operation planning. In addition, eight holes spreading evenly around the tumour region were designed to allow the injection of blue dye for marking the internal tumour resection boundary, as indicated by the green columns in Figure 2c,d. Specifically, the middle point of the longest axis of the tumour was chosen as the centre point of the tumour, based on which a circle was drawn with a diameter slightly extended by 0.5 cm from the MRI tumour boundary to ensure a safe margin [21]. Furthermore, topology optimisation was carried out, and the device was designed with multiple irregular voids to reduce the weight and save cost while maintaining reasonable stiffness. The optimised BSG was generated using the Grasshopper software, and 80 Voronoi cells were randomly distributed to the whole volume of the BSG. To maintain the appropriate stiffness of the part, a constraint that the solid edges between two adjacent voids were larger than 3 mm was applied. Such topology optimisation can reduce the breast tissue’s deformation when the BSG is applied on the breast’s surface, therefore improving tumour marking accuracy. The stiffness was adequate because the BSG was used only for tumour positioning and marking, and there was no large force exerted on it during surgery. Finally, the BSG model was exported in STL format, a standard input format widely used in 3D printing. Each MRI image slice used for 3D reconstruction possessed a layer thickness of less than 3 mm to ensure a relatively fine resolution. The tumour morphology can be accurately sketched; the bone and skin data can also be extracted. The designed BSG model was directly outputted to the STL format in the software with a ratio of 1:1 to preserve the resolution. The thickness of the BSG was designed to be 3 mm to ensure adequate strength.

**Figure 3:**
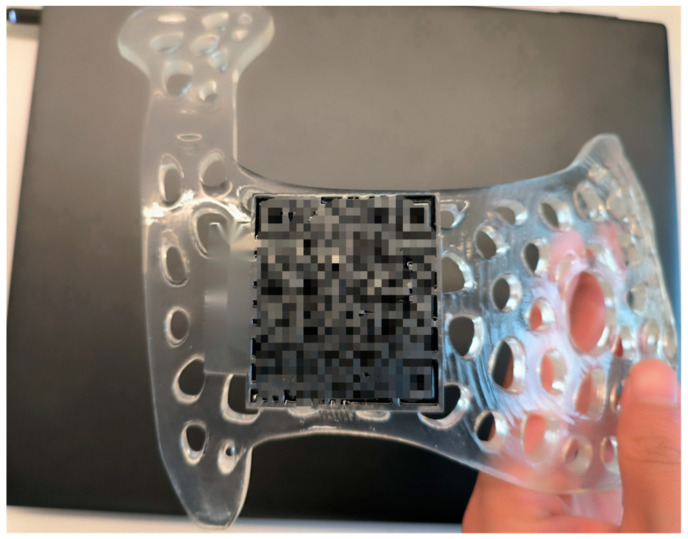
The breast surgery guide fabricated using a stereolithography 3D printer with photocurable material. A patient-specific barcode was attached to store personal information, thus avoiding confusion.

The STL files containing the surface geometric information of the designed BSG were fed into a 3D slicer software for slicing essential for 3D printing. The device was then fabricated using a stereolithography (SLA) 3D printer that creates 3D parts by crosslinking photocurable materials in a layer-wise fashion. Each device was attached with a barcode containing the patient’s personal information (see Figure 3), thus avoiding confusion. Alicyclic epoxy resin (BASF Tinuvin571, BlackFlame Inc., Shanghai, China) composed of alicyclic epoxy compound, epoxy diluent, photoabsorber and photoinitiator (mixed type triarylsulphonium hexafluoroantimonate salts) was used to fabricate the BSG due to its high transparency and excellent heat resistance. The shore hardness, tensile strength and breaking elongation of the solidified specimen are greater than 55 HD, 5 MPa and 20%, respectively, according to the official menu of the product. The Young’s modulus is ~2700 MPa. The material’s desirable ambient printing temperature range is 20–28 °C, at which the rheological properties of the liquid resin are optimal. A 365 nm SLA printer (iSLA550lite, ZRapid Tech, Suzhou, China) with an upside-down mode and an optical resolution of 10 µm was employed for patterning the BSG by photopolymerising the photosensitive material. Specifically, the BSG model in STL format was imported into the slicing software (3dLayer-Standard, 4.2.247, Copyright^©^ ZRapid Tech, Suzhou, China), and a 0.1 mm thin plate was added as the supporting structure. The model was then sliced with a layer thickness of 0.1 mm. Prior to printing, the resin was stirred to ensure it was well mixed and subsequently left for 30 min to remove the bubbles. Following the recommendation of the manufacturer, the light intensity and layer thickness were chosen to be 375 mW cm^−2^ and 0.1 mm, respectively, ensuring that the curing depth was slightly larger than the layer thickness necessary to enhance the bonding of two neighbouring layers while achieving the desired balance between printing speed and vertical resolution. The entire printing was conducted at a constant ambient temperature of 25 °C. After printing, the patterned BSG was immersed in isopropyl alcohol for 15 min to soften the supporting structure for removal. The part was then cleaned using alcohol and stored in a sterile cabinet after drying for further use.

### 2.3. Operation

**Figure 4:**
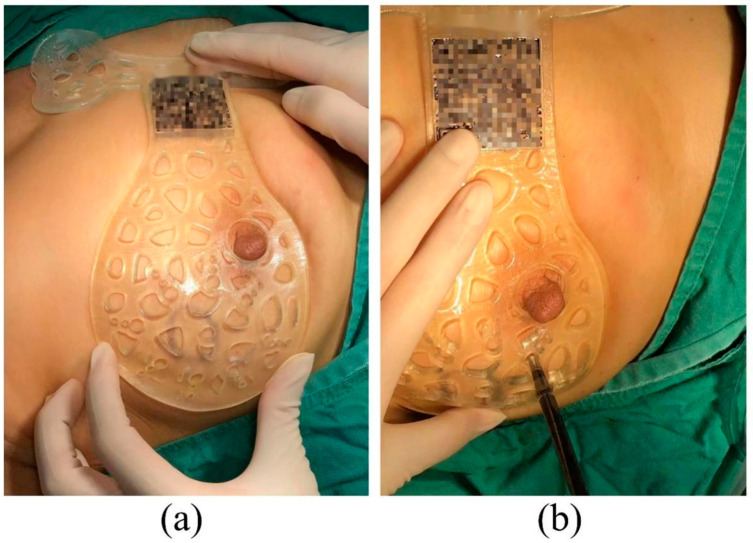
Tumour localisation and margin determination: (**a**) Dual positioning of BSG based on manubrium and nipple to accurately map the tumour area identified on MRI onto the breast directly. (**b**) Blue dye was injected into the breast to mark the internal tumour boundary, guiding the surgeon for tumour resection intraoperatively.

**Figure 5:**
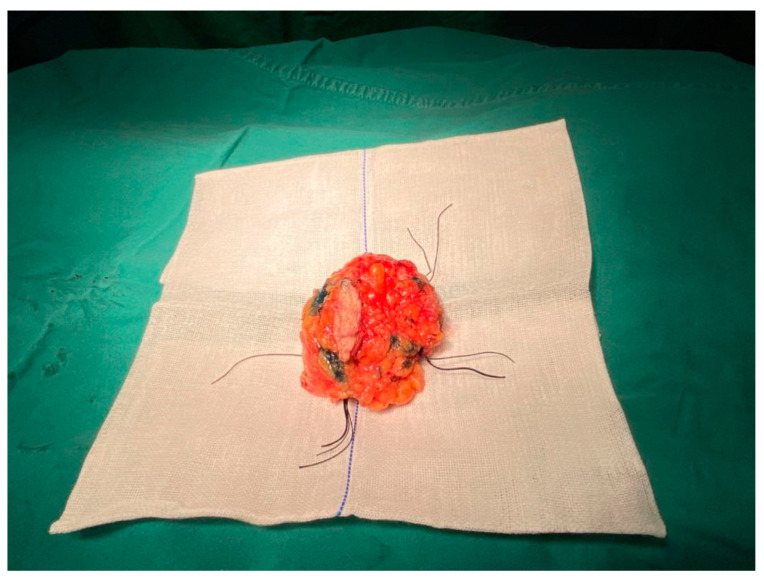
The excised tumour using the BSG-based method. A regular hemispherical-like shape was achieved, showing satisfactory cosmetic outcomes.

After general anaesthesia and sterilisation, the patient’s upper limb on the surgical side was outstretched. The surgical guide plate was placed gently on the breast, where the nipple was inserted into the target hole of the surgical guide to ensure the correct position. Then, the azimuth was adjusted by rotating the device to overlap the patient’s manubrium with that of the surgical guide plate such that the breast and the plate were completely fitted and the projection of the lesion was well overlapped (Figure 4a). The methylene blue dye was injected perpendicular to the surface of the pectoralis major muscle through the predefined eight holes of the device that mark the margin of the tumour (Figure 4b), and each location was injected with approximately 0.3 mL. The dye was injected to the deeper part of the mammary gland to stain the glandular tissue and was thus capable of marking the internal tumour resection boundary. The tumour was then removed by resecting the tissue along the boundary formed by the blue dye (Figure 5). Tissues at the 3, 6, 9 and 12 o’clock directions of the cavity were then taken for frozen pathology to identify whether there was any residual cancer in the resection margin and decide to perform axillary lymph node dissection or not.

### 2.4. Pathological Analysis

The specimens were frozen at −20 °C in a freezer for frozen biopsy pathology analysis. According to the surgeon’s marking points, the material was collected using the method of radial sections perpendicular to the margin. After staining, the samples were placed under the microscope to examine whether there were tumour cells in the cutting margin and to measure the distance between the resection margin and the tumour edge.

Postoperative pathological assessment was conducted to clarify the tumour characteristics such as type, grade and stage. Immunohistochemistry and tumour molecular pathology tests were performed, including oestrogen receptor (ER), progesterone receptor (PR), human epidermal growth factor receptor 2 (HER2) immunohistochemical staining and Ki-67 proliferation index tests.

## 3. Results and Discussion

**Figure 6:**
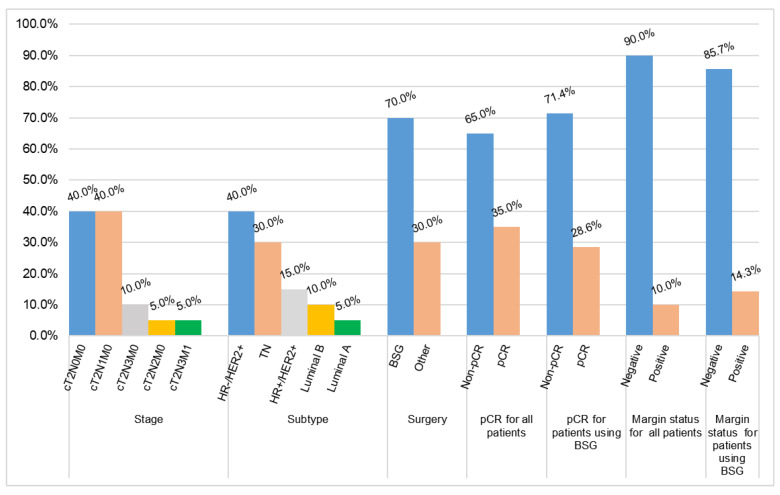
Summary of the preoperative and postoperative pathological characteristics. BSG stands for breast surgical guide. The BCS after NACT was conducted for 20 patients in total, 14 of whom were treated with the BSG-based method.

From January 2018 to July 2023, 20 patients with locally advanced breast cancer were enrolled in this study, and 6 patients refused to use a BSG for BCS following a discussion. Table 1 summarises patients’ information and preoperative tumour characteristics. The median age of the patients was 48, and a higher portion (60%) was below 50 years old. The pathology evaluation revealed that the majority (90%) were invasive ductal carcinoma patients. Four patients (20%) were found to have a positive lymph node, similar to that reported by Wu et al. [21]. For the 14 patients willing to undergo BCS with the BSG a breast MRI examination (prone and supine positions) was performed every two chemotherapy cycles after NACT to evaluate the curative effect. When the condition reached the standard for BCS, the breast and the reduced measurable lesions were modelled using the digital medical design platform (Figure 1 and Figure 2) to provide essential quantitative information for designing a personalised BSG. The device was then manufactured using a 3D printer (Figure 3) and applied for tumour resection (Figure 4). The blue dye staining marks guided the tissue excision within the planning range, and the operation time varied depending on whether or not axillary dissection was performed. The axillary lymph node dissection group was ~80 min and ~55 min for the non-dissection group. The tumour sizes of pre-NACT ranged from 1.4 to 5.7 cm, while they were 0.4–2.5 cm or nondetectable by MRI after NACT, exhibiting significant shrinking. Figure 6 depicts the statistical results of the preoperative and postoperative pathological characteristics, and the raw data are available in Table 2. Among the 14 patients who successfully conducted BCS using a BSG, the pCR rate was ~30% (see Figure 6), similar to that (~30%) reported by Curigliano et al. [25]. Triple negative (TN) and human epidermal growth factor receptor 2 positive (Her-2+) were the main subtypes among all candidates due likely to their high responsiveness to NACT [26]. Two positive margins (~14.3%) were identified via the frozen biopsy, including one TN type and one Her-2+. Negative margins were achieved after the second extended resection, and residual tumour cells were not found during intraoperative freezing biopsy and postoperative pathology evaluation.

Breast cancer is the most common malignant tumour globally [1]. NACT has become an important strategy to downstage tumours, increasing the probability of BCS after NACT in patients with locally advanced breast cancer. However, previous studies [27,28] revealed that the local recurrence rate for patients treated with BCS following NACT was higher than that of adjuvant chemotherapy or primary tumours treated with BCS directly, owing likely to inadequate tumour resection. NACT can cause the initial tumours to shrink dynamically with various patterns, and the tumour is commonly identified using medical imaging methods preoperatively, making intraoperative tumour localisation and margin determination difficult, which can potentially lead to incomplete resection and thus promote local recurrence rate. Wu et al. demonstrated the feasibility and flexibility of the BSG-based method for breast cancer treatment via BCS after NACT, overcoming the shortcomings and limitations existing in traditional localisation approaches [20]. The BSG proposed in this study allows the digital information of the tumour and surrounding breast tissue obtained from imaging tools to be transferred to the designed surgical device by means of image segmentation and 3D reconstruction techniques. The tumour location is then projected precisely from the device onto the surface of the patient’s breast using a dual-positioning method. Meanwhile, the tumour’s internal margin can be marked by injecting colour dye via the dedicated voids on the BSG, offering the surgeon additional guidance for accurate resection. Compared with the traditional localisation methods, such as the wire-guided and clip marker techniques that cause pain and bleeding, wire dislodgment and clip mitigation [15], the localisation using the BSG does not require any traumatic intervention and radio-related guide and is thus less invasive and radioactive-free. The positive margin in this study was ~14.3% (see Table 2), lower than that (20–30%) of the wire-localised tumour excision [19]. In addition, the BSG-based method does not require any imaging guidance and extensive communication and cooperation among the surgeon, radiologist or other specialists during intraoperative localisation necessary generally by the traditional methods, therefore simplifying the surgical process significantly. Furthermore, the operational procedures of the BSG-based methods can be standardised as they do not rely heavily on the skills and experience that vary among surgical staff, promoting the reliability and reproducibility that are of great importance to clinical practice. Finally, the goal of BCS is to remove the tumour with negative resection margins safely while avoiding unnecessary resection of healthy tissue [13]. As shown in Figure 5, the excised tumour based on the BSG exhibited a regular hemispherical-like shape, and no side effects were found to relate to the application of the surgical guide, exhibiting less tissue loss and satisfactory cosmetic outcomes.

Postoperative care is also indispensable. The appearance and dryness of the incision were monitored, and the dressing was changed regularly to prevent infection. The drainage tube was removed by examining the amount and colour of the daily drainage fluid. The pain assessment was conducted for the patient. Psychologists were assigned to patients to take special care of patients’ mental health. The patients were also provided with appropriate dietary advice.

The consistency of the tumour information (location and margin) identified by imaging tools preoperatively and those applied on the patient’s breast intraoperatively is vital for the localisation accuracy of the BSG-based approach. The entire process of this strategy can be divided into three stages: (1) image acquisition; (2) digital tumour information transferred from images to the device; (3) tumour information projection from the device to the patient’s breast. Each step is affected by several factors. During the first stage, the imaging tool selection is crucial for accurately identifying the tumours. MRI has been reported to be the superior imaging modality for assessing the morphology and extent of breast cancer and is widely used for surgical decisions to achieve better negative surgical margins [7,8,29]. However, the tumour-shrinking pattern of NACT is polymorphic, and scattering occurs during the treatment, imposing a huge challenge for capturing lesions with micro sizes [6,30]. The discrepancy in the patient’s posture between MRI assessment and operation can also influence the localisation accuracy because the prone position is generally adopted for obtaining MRI data, while it is supine during the operation [20,21]. This study employed a supine position for MRI to minimise the localisation error. Within the second stage, tumour segmentation essential for designing the BSG is generally performed by radiologists who possess different skills and experience, yielding different results in the tumour extent for the same patient. A larger margin can cause excessive resection and cosmetic issues, while a smaller one may result in a positive margin and thus raise the recurrence rate. Another factor affecting the accuracy is how to decide the resection margin based on the tumour boundary presented on the MRI images. In the current practice, the resection margin is obtained by adding an offset to the MRI boundary, attempting to achieve adequate margins; however, the value of the added width remains controversial [31]. This study extended the MRI boundary by ~0.5 cm (reported by Wu et al. [20]) as the resection margin; two positive margins were present (see Table 2), and negative margins were achieved after the second extended excision. In addition, the 3D-printing modality used to fabricate the surgical guide may also impact the accuracy as they have various printing resolutions [32,33]. This study employed the stereolithography 3D-printing method that has a typical printing resolution of tens of micrometres [16], ensuring high manufacturing precision. For the last stage, accurate positioning of the device on the surface of the breast is essential for precise tumour resection. Unlike the single-point positioning proposed by previous studies [20,21], a dual-positioning design based on manubrium and nipple was presented in this study, allowing the surgeon to fit the device correctly and thus ensuring the tumour location and margin on the device is matched precisely with that of the patient’s breast. Given that the internal boundary is invisible, the injection of blue dye offers visual guidance for tumour resection. The stiffness of the BSG can also impact the positioning accuracy. The BSG fabricated with softer materials is suitable for a more complete adhesion to the breast surface; it is, however, easier to deform, and relative sliding during surgery is more likely to occur. This study adopted a stiffer resin to generate the BSG (Young’s modulus: ~2700 MPa), which can prevent the sliding effectively during the injection of blue dye and therefore mark the tumour margin correctly.

Regarding the limitations of this study, the clinical application cases may be insufficient, as the BSG was applied to 14 patients from January 2018 to July 2023. More operable patients will be enrolled continuously in the future to provide statistical meaning to this study. In addition, tumour segmentation based on MRI data was conducted manually slice-by-slice for each patient, which is not only time-consuming but also lacks consistency. Artificial-intelligence-based segmentation technology might be an optimal alternative to automate this procedure [34].

## 4. Conclusions

Aiming at facilitating the clinical treatment for patients eligible for BCS after NACT, a BSG-based tumour localisation method is proposed in this study and was applied to 14 patients from January 2018 to July 2023. The patient-specific BSG was developed based on supine MRI data, assisted with image segmentation, image 3D reconstruction and 3D-printing technologies, making it possible to project the tumour location and margin identified from MRI onto the patient’s breast precisely, thereby promoting the accuracy of resection and reducing the re-excision rate. The BSG employs a dual-positioning method to improve the positioning accuracy when applied to the breast, while the injection of blue dye offers the surgeon visual guidance for precise tumour resection. Twelve negative resection margins were achieved, accounting for ~85.7%. The excised tumours exhibit a regular hemispherical-like shape, indicating satisfactory cosmetic outcomes. Compared with traditional localisation methods, the BSG-based strategy is less invasive, radiation exposure-free, simpler and possesses a shorter operation time. In addition, it mitigates the dependency on the skills and experience of the surgical team, improving the surgical reliability and procedural standardisation that are important to clinical practice. In the future, more prospective trials are still needed to verify the safety and efficacy of this technology in depth.

## Figures and Tables

**Table 1:** Patient information and tumour characteristics.

Variables		N (%)
Age (years)	Median	48
	Range	26–68
	<50	12 (60.0)
	>50	8 (40.0)
Pathology	Invasive ductal carcinoma	18 (90.0)
	Invasive lobular carcinoma	2 (10.0)
Stage	cT2N0M0	8 (40.0)
	cT2N1M0	8 (40.0)
	cT2N2M0	1 (5.0)
	cT2N3M0	2 (10.0)
	cT2N3M1	1 (5.0)
Subtype	HR+/HER2+	3 (15.0)
	HR-/HER2+	8 (40.0)
	Luminal A	1 (5.0)
	Luminal B	2 (10.0)
	TN	6 (30.0)
Histologic grade	II	12 (60.0)
	III	8 (40.0)
Lymph node status	Positive	4 (20.0)
	Negative	16 (80.0)

**Table 2:** Preoperative and postoperative pathological characteristics for patients eligible for BCS. IIC and IDC express invasive lobular carcinoma and invasive ductal carcinoma, respectively. BSG indicates that the tumour resection is performed using the breast surgery guide. Note that ductal carcinoma in situ was found for patient 20.

Patient ID	Pathology (IIC or IDC)	Stage	IHC	Subtype	Histologic Grade	Lymph Node Status	Neoadjuvant Therapy	Margin Status	pCR	Surgery Type
1	IIC	cT2N0M0	ER (−), PR (−), HER-2(3+), Ki67(10%)	HR-/HER2+	II	Negative	TCbHP X6	Negative	Non-pCR	BSG
2	IDC	cT2N0M0	ER(3+ 90%), PR(2+ 70%), HER-2(1+), Ki67(20%)	Luminal A	II	Negative	AC-T X8	Negative	Non-pCR	BSG
3	IDC	cT2N2M0	ER(3+ 30%), PR(−), HER-2(3+), Ki67(50%)	HR+/HER2+	III	Negative	THP X5	Negative	Non-pCR	BSG
4	IDC	cT2N0M0	ER(−), PR(−), Her2(2+), Ki67(40%+)	HR-/HER2+	III	Negative	TCbH X6	Negative	Non-pCR	Other
5	IIC	cT2N1M0	ER(2+ 80%), PR(3+ 80%), Her2(3+), Ki67(40%)	HR+/HER2+	II	Negative	AC-THP X8	Positive	Non-pCR	BSG
6	IDC	cT2N0M0	ER(−), PR(2+ 50%), Her2(0), Ki67(10%)	Luminal B	II	Negative	TAC X6	Negative	Non-pCR	BSG
7	IDC	cT2N0M0	ER(−), PR(−), Her2(0), Ki67(35%)	TN	III	Negative	TAC X6	Negative	pCR	Other
8	IDC	cT2N0M0	ER(−), PR(−), Her2(0), Ki67(30%)	TN	II	Negative	TAC X6	Negative	Non-pCR	BSG
9	IDC	cT2N1M0	ER(−), PR(−), Her2(1+), Ki67(+50%)	TN	III	Negative	TAC X6	Negative	Non-pCR	BSG
10	IDC	cT2N1M0	ER(−), PR(−), Her2(3+), Ki67(30%)	HR-/HER2+	III	Negative	TCbHP X6	Negative	pCR	BSG
11	IDC	cT2N1M0	ER(−), PR(−), Her2(3+), Ki67(30%)	HR-/HER2+	II	Negative	TCbHP X6	Negative	pCR	BSG
12	IDC	cT2N1M0	ER(−), PR(−), Her2(3+), Ki67(30%+)	HR-/HER2+	II	Negative	TCbHP X6	Negative	pCR	BSG
13	IDC	cT2N3M0	ER(−), PR(−), Her2(2+), Ki67(+ 10%)	TN	III	Positive	TAC X6	Negative	pCR	Other
14	IDC	cT2N3M1	ER(−), PR(−), Her2(3+), Ki67(10%)	HR-/HER2+	II	Negative	TCbHP X6	Negative	pCR	Other
15	IDC	cT2N1M0	ER(2+ 90%), PR(2+), Her2(1+), Ki67(+ 10%)	Luminal B	II	Positive	TAC X6	Negative	Non-pCR	Other
16	IDC	cT2N0M0	ER(+5%), PR(+5%), Her2(3+), Ki67(30%)	HR+/HER2+	III	Negative	TCbHP X6	Negative	Non-pCR	Other
17	IDC	cT2N1M0	ER(-), PR(−), Ki67(+50%), Her2(1+)	TN	II	Negative	AC-T X8	Negative	Non-pCR	BSG
18	IDC	cT2N3M0	ER (−), PR (−), Her2 (3+), Ki67(20%)	HR-/HER2+	III	Positive	TCbHP X6	Negative	Non-pCR	BSG
19	IDC	cT2N0M0	ER(+), PR(−), Ki67(50%+), Her2(3+)	HR-/HER2+	II	Negative	AC-TH X6	Negative	Non-pCR	BSG
20	IDC	cT2N1M0	ER(−), PR(−), HER-2(2+), Ki67(20%+)	TN	II	Positive	AC-T X8	Positive	pCR	BSG

## Data Availability

The data that support the findings of this study are available from the corresponding author upon reasonable request.
